# Thickness of the bone-cartilage unit in relation to osteoarthritis severity in the human hip joint

**DOI:** 10.1136/rmdopen-2018-000747

**Published:** 2018-09-21

**Authors:** Louise Brøndt Hartlev, Rasmus Klose-Jensen, Jesper Skovhus Thomsen, Jens Randel Nyengaard, Lene Warner Thorup Boel, Mogens Berg Laursen, Trine Bay Laurberg, Andreas Wiggers Nielsen, Kristian Steengaard-Pedersen, Ellen-Margrethe Hauge

**Affiliations:** 1 Department of Rheumatology, Aarhus University Hospital, Aarhus, Denmark; 2 Department of Biomedicine – Anatomy, Aarhus University, Aarhus, Denmark; 3 Department of Clinical Medicine, Core Centre for Molecular Morphology, Section for Stereology and Microscopy, Centre for Stochastic Geometry and Advanced Bioimaging, Aarhus University, Aarhus, Denmark; 4 Institute of Forensic Medicine, Aarhus University, Aarhus, Denmark; 5 Orthopaedic Surgery Research Unit, Aalborg Hospital – Aarhus University Hospital, Aalborg, Denmark; 6 Department of Clinical Medicine, Aarhus University, Aarhus, Denmark

**Keywords:** osteoarthritis, subchondral bone, stereology, articular cartilage, calcified cartilage, hip

## Abstract

**Objective:**

Bone formation is a hallmark of osteoarthritis (OA). It has been speculated that bone formation may occur because of ossification at the bone-cartilage unit, that is, bone formation directly involving the calcified cartilage (CC). This study aimed to investigate the thickness of the CC and subchondral bone (SCB) in relation to the severity of the overlying articular cartilage (AC) degeneration.

**Design:**

We investigated femoral heads from 20 patients with OA and 15 healthy subjects with design-based stereology using systematic uniform random sampling of the entire joint surface. This was combined with the Osteoarthritis Research Society International (OARSI) OA cartilage histopathology assessment system, thus obtaining focal OARSI grades paired with thickness measurements of AC, CC and the SCB.

**Results:**

The patients with OA had thicker CC (mean 159; 95% CI 144 to 177 µm) compared with the healthy subjects (mean 132; 95% CI 113 to 1550 µm; p=0.036), and this difference was even higher in areas without loss of AC thickness (OARSI grade ≤3); 187 (95% CI 164 to 214) µm vs 132 (95% CI 113 to 155) µm (p=0.001). In the patients with OA, a thicker SCB was observed in areas with loss of AC thickness (OARSI grade ≥4), but not in areas without loss of AC thickness (OARSI grade ≤3).

**Conclusion:**

The study showed that thicker CC is present in early stages of OA, suggesting that bone formation by endochondral ossification is an early phenomenon of OA. Thickening of the SCB was present, but only in areas with denuded bone. Suggesting that also appositional bone growth occurs and that it may be a consequence of changed biomechanics.

**Key messages**

**What is already known about this subject?**

- Alterations in the composition, functional properties and structure of the bone-cartilage unit occur during the development of osteoarthritis (OA).

**What does this study add?**

- Calcified cartilage thickening is found, where no loss of articular cartilage has occurred, suggesting endochondral ossification to be an early phenomenon in OA.
- Subchondral bone thickening is strongly associated with loss of articular cartilage and appears to be a late-stage phenomenon caused by appositional growth.

**How might this impact on clinical practice?**

- Possible disease-modifying interventions focused at the bone-cartilage unit might target different processes relevant for early or late OA disease stages.

## Introduction

Hip osteoarthritis (OA) is not merely a disease of ‘wear and tear’ of the articular cartilage (AC).^1^ Bone formation is a hallmark of OA and may be part of the pathophysiology.^2^ Most studies of OA have focused separately on the AC,^3^ the subchondral bone (SCB)^2^ or other joint tissues.^4^ Few studies have investigated the association between the tissues and their correlation with the degree of focal cartilage loss.^5 6^

It has been suggested that the thin layer of calcified cartilage (CC) between the AC and the SCB plate plays a role in the communication between the bone and cartilage in patients with OA.^7^ Moreover, it has been suggested that the CC may be involved in endochondral ossification leading to bone formation in OA.^8–10^ Bone growth in OA has been confirmed by several studies, which found that the thickness of the SCB plate increased,^5 11–15^ in addition to the formation of osteophytes in OA.^16–18^ It remains, however, to be shown whether the growth of the SCB plate can be associated with either bone apposition or endochondral ossification. If bone growth occurs as a consequence of endochondral ossification, as have been suggested,^19 20^ then the tidemark would advance into the AC leading to a thickening of the CC and thinning of the AC. In contrast, bone apposition at the lower aspect of the SCB plate by modelling does not involve the CC.^21^ Therefore, a characterisation of the CC is essential in order to understand how the thickness of the SCB plate occurs during development and progression of OA.

Currently, it is difficult to investigate the CC in human OA, since the CC cannot be identified in vivo by either conventional X-ray, CT or MRI. The CC, therefore, needs to be investigated using other methods, for example, histology.

The study aimed to investigate the thickness of the SCB and the CC and their relation to the different severity of AC damage in entire femoral heads from both patients with OA and healthy subjects. AC damage was determined by morphometric assessment of grade and stage (cf Osteoarthritis Research Society International (OARSI) scoring method).^22^ We hypothesise that the CC and SCB plate thickness increase progressively with cartilage damage in patients with OA.

## Materials and methods

### Study population

Human femoral heads from 35 subjects comprising 20 patients with OA with a mean age of 63.6 years (range 58.9–68.3 years) and 15 healthy subjects with a mean age of 64.3 years (range 60.5–68.0 years) (table 1) were included in the study.^5^

**Table 1:** Characteristics of healthy subjects and patients with osteoarthritis

	Healthy subjects (n=15)	Patients with osteoarthritis (n=20)
Women:men (n)	7:8	10:10
Age (years)	63.6 (58.9 to 68.3)	64.3 (60.5 to 68.0)
Weight (kg)	80.6 (66.4 to 94.8)	79.8 (73.2 to 86.5)
Height (cm)	173.7 (168.0 to 177.3)	170.2 (166.0 to 174.0)
BMI (kg/m^2^)	26.8 (22.8 to 30.8)	27.5 (25.5 to 29.5)
Kellgren-Lawrence grade	–	3.9 (3.8 to 4.0)
WOMAC score		
Pain	–	49.9 (39.4 to 60.3)
Stiffness	–	58.2 (45.2 to 71.2)
Physical activity	–	49.9 (33.2 to 47.1)

Data are presented as mean and (95% CI).

BMI, body mass index; WOMAC, Western Ontario and McMaster Universities Arthritis Index.

Twenty femoral heads were obtained from patients with primary hip OA, who underwent hip replacement surgery at the Department of Orthopaedics at Farsoe Hospital in Denmark. The patients met the combined clinical and radiographic criteria of the American College of Rheumatology for OA.^16^ Patients with known bone metabolic diseases, secondary OA or other joint diseases, diabetes mellitus or malignant diseases were excluded from the study.

The femoral heads from the control group were obtained at autopsy from healthy subjects with macroscopically normal femoral heads. All healthy subjects had died suddenly from accidents or acute diseases. Subjects with a history of high-energy pelvic trauma or any signs of hip OA after macroscopic inspection were excluded. Furthermore, healthy subjects were excluded if they had any known diagnosis of bone metabolic disease, other joint diseases, diabetes mellitus or malignant diseases.

### Processing of tissue

**Figure 1:**
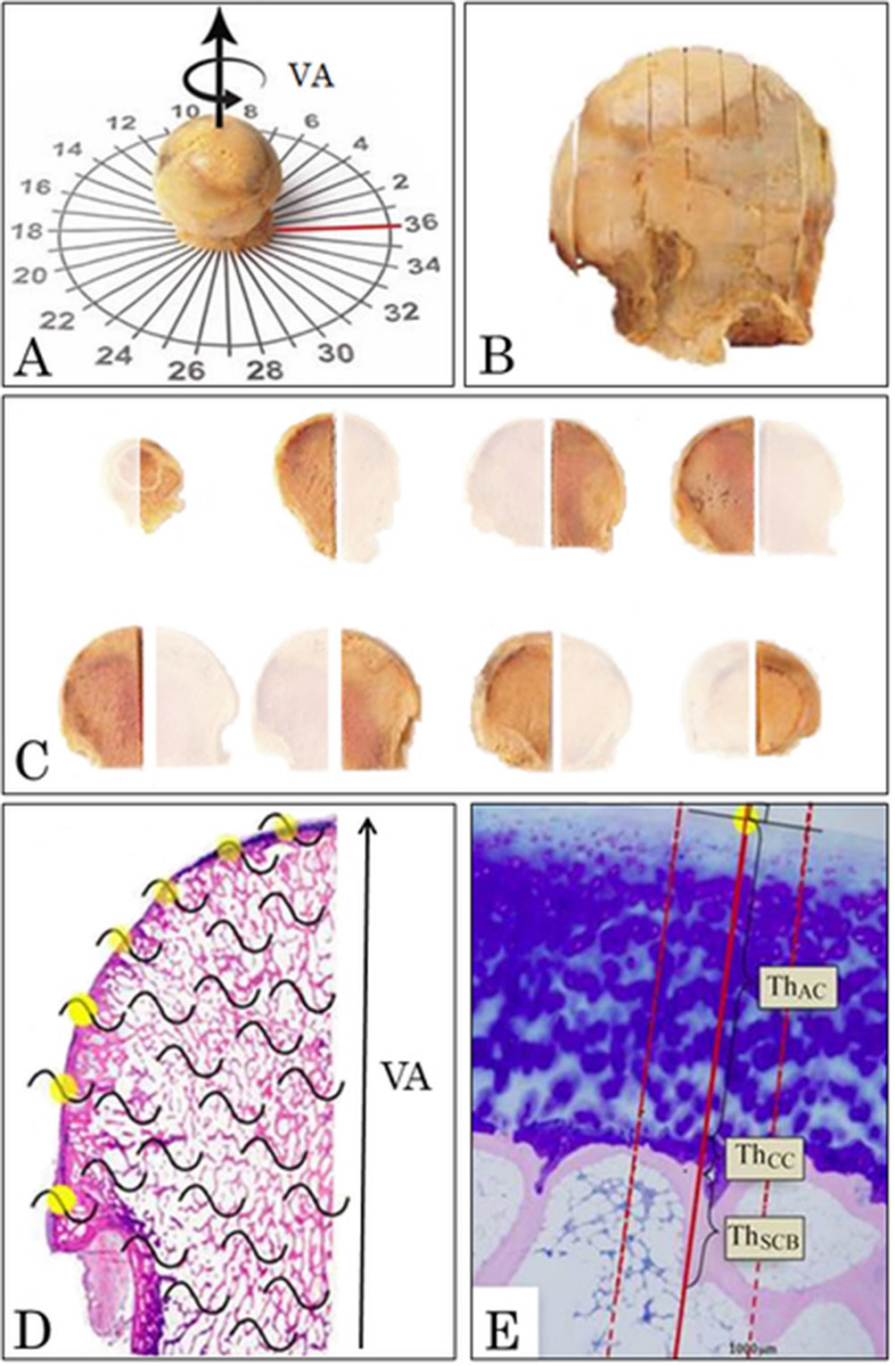
Design-based stereological methods using systematic uniform random sampling and vertical sections were used to study the undecalcified human femoral heads. (A) The vertical arrow represents the vertical axis (VA) of the femoral head and the rotated arrow represents the random rotation of the femoral head around the VA. The red line indicates the location of the medial part of the femoral head. (B) The femoral head was cut in 7 mm thick slices in a random orientation and parallel to the VA to get vertical uniform random sections. (C) The 7 mm thick slices were halved and alternating left and right half slices were sampled randomly resulting in a sampling fraction of one-half. (D) From each femoral head, all the sampled 7 mm thick halved parallel slices were embedded in methylmethacrylate, and 7 µm thick histological sections were cut from each slice and stained with May-Grünwald toluidine blue. In all histological sections, the joint surface was sampled systematically uniform random as every intersection (yellow dots) between the superimposed cycloid grids (black curved lines) and the joint surface. (E) For each random sampling point, the Osteoarthritis Research Society International (OARSI) grade was determined, and a line was drawn perpendicular to the joint surface. Along the line drawn perpendicular to the joint surface, the Th_AC_, Th_CC_ and Th_SCB_ were measured. To increase the precision of the Th_SCB_, two lines separated by 600 µm were drawn parallel to and on each side of the line perpendicular to the joint surface. The subchondral bone (SCB) plate thickness was also measured along these additional two lines, and the average was used as the estimate of the SCB plate thickness at that specific sampling point. AC, articular cartilage; CC, calcified cartilage.

Immediately after removal of the femoral heads, they were fixated in 70% ethanol and processed according to a design-based stereological sampling scheme as previously described in detail.^23^ In brief: the entire femoral heads were rotated around a vertical axis (VA), which was perpendicular to the anatomical top of the femoral head (figure 1A). After choosing a random starting point, the femoral head was sawed using a diamond precision-parallel saw (Exakt Apparatebau, Norderstedt, Germany) into 7 mm thick parallel slices (figure 1B), which were halved, and alternating left and right half slices were randomly selected for the following microscopic evaluation^23 24^ (figure 1C). Depending on the size of the femoral head, a total of five to seven 7 mm thick halved parallel slices were collected from both patients with OA and healthy subjects. Each of the five to seven 7 mm thick halved parallel slices was embedded undecalcified in methylmethacrylate and cut into 7 µm thick histological sections using a Jung model K microtome (R Jung, Heidelberg, Germany) equipped with a tungsten microtome knife. The sections were mounted and stained with May-Grünwald toluidine blue as previously described^23^ (figure 1D).

### Microscopy and sampling

The entire femoral head surface area was estimated by systematic uniform random sampling^25^ and vertical uniform random sections (figure 1).^24^ For each of the femoral heads, a total of 50–70 random sampling points were generated by superimposing a cycloid line grid (area per length, a(*l*)*=*2 mm) onto each of the five to seven 7 µm thick sections. The random sampling points were the intersections between the joint surface and the test lines of the cycloid line grid (figure 1D).^26^ For each random sampling point, the OARSI grade was determined using the OARSI scoring method described by Pritzker *et al*.^22^ From all random sampling points, a line was drawn perpendicular to the joint surface, and along this line, the thickness of the AC, CC and SCB plate was measured (figure 1E). In order to increase the precision of the SCB plate thickness estimates, a parallel line was drawn on either side of the line perpendicular to the joint surface (figure 1E). These two lines were both separated by 300 µm from the perpendicular line. For each random sampling point, the SCB plate thickness was measured along these three lines, and the average was used (figure 1E). Fovea capitis and osteophytes were excluded from the analysis. All measurements were performed by one observer, who was blinded for the group distribution.

The sections were evaluated under a light microscope (Olympus BH-2, Tokyo, Japan), equipped with a projection arm and a digitiser tablet (Wacom Intuos-3, Wacom, Otone, Japan) attached to a PC with SigmaScan Pro-5 (Systat Software, San Jose, CA) and a computer mouse mounted with a point-like red LED. The sections were analysed at a magnification of ×31.

### OARSI grading, staging and scoring

The OARSI grade (range 0–6) was determined according to the guidelines by Pritzker *et al*, where grading is based on structural and vertical changes of the joint surface which includes normal cartilage (grade 0), cartilage fibrillation (grade 1), discontinuity of the cartilage surface (grade 2), fissuring (grade 3), erosion and loss of cartilage thickness (grade 4), denudation of the surface (grade 5) and lastly deformation of the bone (grade 6).^22^ The OARSI stage (range 0–4) describes the horizontal extent of the articular joint surface irrespective of the underlying OARSI grade.^22^ The OARSI score was determined according to the recommended OA scoring method proposed by Pritzker *et al*. The OARSI score has a range of 0–24 based on the most advanced grade and most extensive stage present.^22^

### Thickness measurements

Corresponding measurements of the OARSI grade, the thickness of the AC (Th_AC_), the CC (Th_CC_) and the SCB (Th_SCB_) were made for each of the 50–70 random sampling points over the entire joint surface of each femoral head (figure 1).

The Th_AC_ was measured as the orthogonal distance from the surface of the AC to the first encounter of the tidemark, while the Th_CC_ was measured from the proximal tidemark to the cement line (figure 1C). The term SCB is ambiguous.^27^ In this study, the SCB plate was defined as the distance from the cement line until the first distal occurrence of bone marrow (figure 1C).^28^

### Thickness correction

In order to fulfil the criteria of systematic uniform random sampling and vertical uniform random sections, the 7 mm thick halved parallel slices were sawed parallel to the VA.^24^ Hence, the measured thicknesses of the tissue structures were obtained perpendicular to the surface in the sawing plane, but not perpendicular to the curved three-dimensional joint surface. Therefore, each of the thickness measurements was corrected for oblique sectioning according to the location of the sawing plane. For full detail of the thickness correction, see online supplementary material.

10.1136/rmdopen-2018-000747.supp1 Supplementary data

### Coefficient of variance

The variance of the applied method and the variance between femoral heads were evaluated using the coefficient of variance of the method (CV_Method_) and the total coefficient of variance (CV_Total_).^29^ CV_Method_ ranged from 0.01 to 0.09, while CV_Total_ ranged from 0.23 to 0.46. Consequently, the variation observed could be attributed to the biological variation between the study subjects rather than the variance of the method.^30^

### Statistics

Data were analysed using STATA V.12 (StataCorp, College Station, TX, USA). Normal distribution of the data was investigated with Q-Q plots and histograms. Normally distributed data were presented as arithmetic mean (95% CI). Data not being normally distributed were log-transformed, checked for normality and presented as geometric mean (95% CI). The geometric mean difference between patients with OA and healthy subjects was found using bootstrapping with bias corrected and accelerated intervals to get 95% CI. Student’s t-test was used to test for statistical significance between the patients with OA and the healthy subjects. The difference of thickness measurement was made for the complete femoral head (OARSI grades 0–6), for areas without loss of cartilage thickness (OARSI≤3) and for areas with loss of cartilage thickness (OARSI grade ≥4). Student’s t-test was also used for comparison between patients with OA and the healthy subjects regarding the thickness measurements for each individual OARSI grade. One-way analysis of variance (ANOVA) was used to investigate the difference of the thickness measurements according to OARSI grades in both patients with OA and healthy subjects. The post hoc Bonferroni test was used to identify specific differences in the thickness measurements between corresponding OARSI grades. The results were considered significant at p<0.05.

## Results

In the patients with OA, the hip X-ray showed a mean Kellgren-Lawrence grade^31^ of 3.9 (3.8–4). The patients with OA had a mean Western Ontario and McMaster Universities Arthritis Index (WOMAC)^32^ score for pain of 49.9 (39.4–60.3), for stiffness of 58.2 (45.2–71.2) and physical activity of 49.9 (33.2–47.1). Neither the WOMAC score nor the Kellgren-Lawrence grade could be obtained from the healthy subjects (table 1).

### OARSI grading, staging and scoring

The mean number of random sampling points of the entire femoral head surface area did not differ between the patients with OA and the healthy subjects (table 2). The mean OARSI grade, stage and score for the entire femoral head were significantly higher for patients with OA compared with the healthy subjects (table 2).

**Table 2:** Comparison of OARSI and thickness measurements of articular cartilage, subchondral bone plate and calcified cartilage between healthy subjects and patients with osteoarthritis

	Healthy subjects		Patients with osteoarthritis		Δ Mean	P values
	n	Mean (95% CI)	n	Mean (95% CI)		
OARSI evaluation						
Random surface sampling points (n)*	15	61 (54 to 68)	20	59 (54 to 65)	2 (−7 to 10)	0.695
OARSI grade	15	0.2 (0.1 to 0.4)	20	2.4 (92.1 to 2.8)	2.2 (1.8 to 2.6)	<0.001
OARSI stage*	15	2.1 (1.7 to 2.6)	20	4.0 (4.0 to 4.0)	1.9 (1.5 to 2.2)	<0.001
OARSI score	15	4.3 (2.7 to 6.9)	20	20.9 (19.3 to 22.6)	16	<0.001
Morphometric assessment of stage (%)						
OARSI grade 0	15	67 (48 to 94)	7	8 (3 to 24)	−61 (−75 to 46)	<0.001
OARSI grade 1	15	15 (10 to 23)	20	21 (14 to 31)	7 (−1 to 16)	0.271
OARSI grade 2	8	5 (3 to 11)	20	25 (21 to 30)	19 (11 to 26)	<0.001
OARSI grade 3	5	6 (2 to 19)	20	12 (9 to 17)	8 (2 to 13)	0.073
OARSI grade 4	–		20	11 (8 to 14)	–	–
OARSI grade 5	–		17	8 (6 to 12)	–	–
OARSI grade 6	–		10	9 (5 to 16)	–	–
Thickness (µm)						
Articular cartilage (Th_AC_)						
OARSI grades 0–6	15	1425 (1310 to 1549)	20	1116 (976 to 1277)	−277 (−443 to 111)	0.003
OARSI grade ≤3†	15	1425 (1310 to 1551)	20	1464 (1323 to 1620)	58 (−159 to 275)	0.564
OARSI grade ≥4‡	–		20	264 (182 to 383)	–	–
OARSI grade 0*	15	1448 (1325 to 1570)	7	1640 (1243 to 2037)	193 (−93 to 478)	0.297
OARSI grade 1	15	1381 (1160 to 1645)	20	1525 (1350 to 1722)	135 (−195 to 464)	0.317
OARSI grade 2	8	1523 (1163 to 1995)	20	1441 (1259 to 1650)	−91(−606 to 423)	0.664
OARSI grade 3*	5	1251 (786 to 1717)	20	1265 (1080 to 1450)	14 (−391 to 419)	0.944
OARSI grade 4*	–		20	584 (437 to 731)	–	–
OARSI grade 5	–		6	56 (14 to 224)	–	–
OARSI grade 6	–		–		–	–
Subchondral bone plate (Th_SCB_)						
OARSI grades 0–6	15	248 (185 to 340)	20	376 (318 to446)	103 (30 to 176)	0.019
OARSI grade ≤3†	15	276 (225 to 339)	20	252 (205 to 308)	−24 (−109 to 62)	0.515
OARSI grade ≥4*‡	–		20	665 (550 to 781)	–	–
OARSI grade 0	15	270 (215 to 339)	7	216 (123 to 381)	−28 (−204 to 148)	0.326
OARSI grade 1	15	248 (188 to 328)	20	200 (151 to 266)	−48 (−175 to 80)	0.272
OARSI grade 2*	8	236 (54 to 418)	20	275 (228 to 322	39 (−82 to 162	0.636
OARSI grade 3	5	307 (140 to 220)	20	314 (252 to *391*)	−15 (−158 to 187)	0.925
OARSI grade 4	–		20	446 (355 to 559)	–	–
OARSI grade 5*	–		17	875 (757 to 992)	–	–
OARSI grade 6*	–		10	870 (691 to 1049)	–	–
Calcified cartilage (Th_CC_)						
OARSI grades 0–6	15	132 (113 to 155)	20	159 (144 to 177)	26 (6 to 45)	0.036
OARSI grade ≤3†	15	132 (113 to 155)	20	187 (164 to 214)	57 (23 to 91)	0.001
OARSI grade ≥4‡	–		20	93 (71 to 122)	–	–
OARSI grade 0	15	131 (112 to 152)	7	153 (111 to 209	24 (−30 to 790	0.269
OARSI grade 1	15	111 (77 to 160)	20	202 (168 to 242)	82 (28 to 1307)	0.005
OARSI grade 2	8	186 (129 to 269)	20	174 (145 to 208)	−17 (−113 to 78	0.703
OARSI grade 3*	5	177 (137 to 219)	20	183 (144 to 223)	6 (−74 to 85)	0.815
OARSI grade 4	–		20	145 (125 to 168)	–	–
OARSI grade 5	–		13	65 (37 to 113)	–	–
OARSI grade 6	–		3	189 (1 to 583)	–	–

Data are presented as geometric mean and (95% CI). Statistical significance was found using Student’s t-test. P<0.05 was considered significant.

*Data that were normally distributed without being log transformed are presented as arithmetic mean (95%CI).

†OARSI grade ≤3 represents areas without loss of articular cartilage thickness.

‡OARSI grade ≥4 represents areas with loss of articular cartilage thickness.

AC, articular cartilage; CC, calcified cartilage; OARSI, Osteoarthritis Research Society International; SCB, subchondral bone.

Applying morphometric staging, the patients with OA had a mean of 18% (11%–21%) of the femoral head surface area with complete loss of AC (OARSI grade ≥5). The femoral head of the healthy subjects, however, was covered entirely by AC of normal thickness (OARSI grade ≤3). In patients with OA, 72% (65%–80%) of the femoral head was covered by cartilage of normal thickness (OARSI grade ≤3).

### Thickness of AC

The patients with OA had statistically significantly lower mean Th_AC_ for the total joint surface area (OARSI grades 0–6) compared with healthy subjects (table 2). There was no significant difference for Th_AC_ between patients with OA and healthy subjects in areas without loss of cartilage thickness (OARSI grade ≤3) (table 2). For patients with OA, Th_AC_ for areas with loss of cartilage thickness (OARSI grade ≥4) was significantly thinner compared with Th_AC_ for areas without loss of cartilage thickness (OARSI grade ≤3) (p<0.001). According to individual OARSI grades, there was no significant difference in AC thickness between the patients with OA and healthy subjects (table 2).

**Figure 2:**
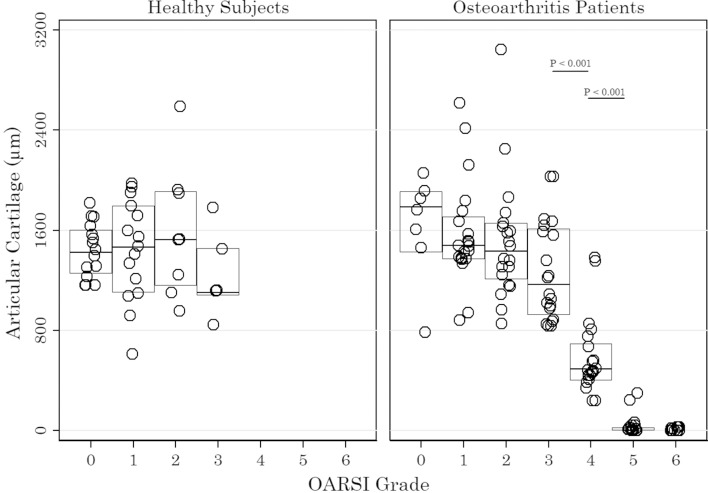
The thickness of the articular cartilage for healthy subjects and patients with osteoarthritis (OA). Each circle represents the articular cartilage thickness categorised according to the OARSI grades 0–6 in an individual. The horizontal line indicates the median while the IQR is represented by the boxes. One-way analysis of variance for Th_AC_ according to OARSI grade was significant for the patients with OA (*F*(5, 87)=62.06, p<0.001) but not for healthy subjects (*F*(3, 39)=0.82, p=0.0491). The p values indicated are the result of post hoc Bonferroni test. P<0.05 was considered significant. OARSI, Osteoarthritis Research Society International.

One-way ANOVA for Th_AC_ according to OARSI grade was significant for the patients with OA (*F*(5, 87)=62.06, p<0.001) but not for healthy subjects (*F*(3, 39)=0.82, p=0.491). The mean Th_AC_ in patients with OA decreased significantly from OARSI grades 3–5 (figure 2).

### Thickness of SCB

**Figure 3:**
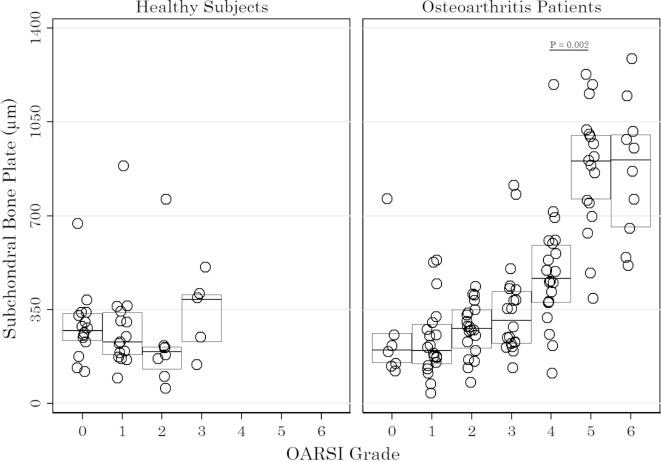
The thickness of the subchondral bone plate for healthy subjects and patients with osteoarthritis (OA). Each circle represents the subchondral bone plate thickness categorised according to the OARSI grades 0–6 in an individual. The horizontal line indicates the median while the IQR is represented by the boxes. One-way analysis of variance for Th_SCB_ according to OARSI grade was significant for the patients with OA (*F*(6, 107)=23.23, p<0.001) but not for healthy subjects (*F*(3, 39)=1.34, p=0.274). The p values indicated are the result of post hoc Bonferroni test. P<0.05 was considered significant. OARSI, Osteoarthritis Research Society International.

The patients with OA had statistically significantly higher mean Th_SCB_ for the total joint surface area (OARSI grades 0–6) compared with healthy subjects (table 2). The Th_SCB_ for areas without loss of cartilage thickness (OARSI grade ≤3) was not significantly different for patients with OA compared with healthy subjects (table 2). According to individual OARSI grades, there was no significant difference in SCB thickness between the patients with OA and healthy subjects (table 2). For patients with OA, Th_SCB_ for areas with loss of cartilage thickness (OARSI grade ≥4) was significantly thicker compared with Th_SCB_ for areas without loss of cartilage thickness (OARSI grade ≤3) (p<0.001). One-way ANOVA for Th_SCB_ according to OARSI grade was significant for the patients with OA (*F*(6, 107)=23.23, p<0.001) but not for healthy subjects (*F*(3, 39)=1.34, p=0.274). The higher mean Th_SCB_ of patients with OA was seen only in areas with total loss of AC, that is, denuded bone surface (OARSI grades 5–6) (figure 3).

### Thickness of CC

**Figure 4:**
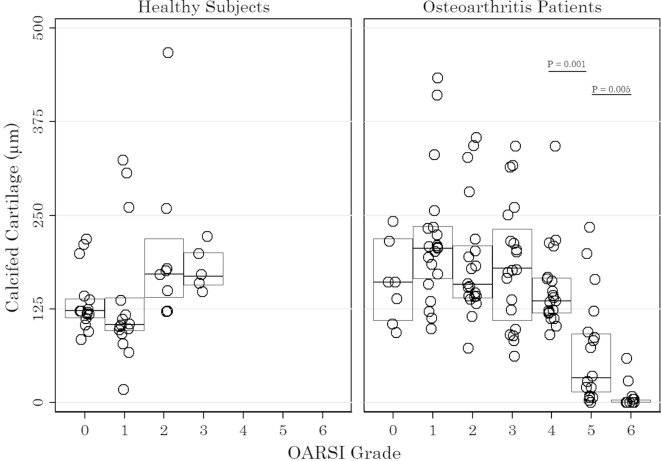
The thickness of the calcified cartilage for healthy subjects and patients with osteoarthritis (OA). Each circle represents the calcified cartilage thickness categorised according to the OARSI grades 0–6 in an individual. The horizontal line indicates the median while the IQR is represented by the boxes. One-way analysis of variance for Th_CC_ according to OARSI grade was significant for the patients with OA (*F*(6, 96)=14.5, p<0.001) but not for healthy subjects (*F*(3, 39)=2.61, p=0.065). The p values indicated are the result of post hoc Bonferroni test. P<0.05 was considered significant. OARSI, Osteoarthritis Research Society International.

The mean thickness of the CC for the total joint surface area (OARSI grades 0–6) was significantly higher for patients with OA compared with the healthy subjects (table 2). For areas without loss of cartilage thickness (OARSI grade ≤3), this difference was even greater between the patients with OA and the healthy subjects (table 2). According to individual OARSI grades, there was significant difference in CC thickness between the patients with OA and healthy subjects at OARSI grade 1 (table 2). For patients with OA, the Th_CC_ in areas with loss of cartilage (OARSI grade ≥4) was significantly thinner compared with Th_CC_ for areas without loss of cartilage (OARSI grade ≤3) (p=0.001). One-way ANOVA for Th_CC_ according to OARSI grade was significant for the patients with OA (*F*(6, 96)=14.5, p<0.001) but not for healthy subjects (*F*(3, 39)=2.61, p=0.065). The mean Th_CC_ in patients with OA decreased significantly from OARSI grade 4 to grade 6 (figure 4).

## Discussion

This study is the first to investigate the thickness of the different tissues in the bone-cartilage unit and their relation to cartilage damage using entire human femoral heads and design-based stereological sampling. The study confirmed that the CC was thicker in patients with OA compared with healthy subjects and that this was present at early stages without loss of AC. The thickening of the SCB plate, however, occurred only in areas where the AC was completely lost.

### Subchondral bone

It is well known that OA is characterised by thickening of the SCB plate.^2 14 15^ In the present study, however, we found that the thicker SCB plate in patients with OA only occurred in areas with denuded bone surface (OARSI grades 5–6) where the SCB plate was more than twofold thicker compared with areas with OARSI grade 4. This is in accordance with our previous study, which found that the SCB volume was higher in patients with OA compared with healthy subjects but only in areas with denuded bone surface.^5^ Therefore, severe bone thickening in areas with denuded bone surface is probably also a result of increased biomechanical load and a reactive increase in bone modelling in favour of bone formation by appositional growth.^21^ This is supported by studies showing increased bone turnover^5^ of hypomineralised^14 33^ and severely thickened SCB in OA.^34^

### Calcified cartilage

The patients with OA had significantly thicker CC compared with the healthy subjects, which was observed in areas without loss of cartilage thickness (OARSI grade ≤3). A thicker CC in early OA indicates an early advancement of the CC into the AC and is suggestive of bone growth by endochondral ossification,^8^ which can lead to the replacement of the AC by bone.^35^

Only a few studies have measured the thickness of the CC in patients with OA. Revell *et al* showed no difference in human patellae between patients with OA and healthy subjects.^33^ The different outcome, by Revell *et al*, can be explained by location, smaller sample size, and that they sampled the central patella only, whereas we applied an unbiased random sampling procedure to measure the thickness of the entire femoral head joint surface, that is, including areas of all OARSI grades. Other studies have shown patients with OA to have a larger tidemark area in the patella compared with the healthy subjects^36^ and increased mineralisation of the AC,^37^ suggesting an increased metabolic activity of the CC in OA, which is in line with our findings in the present study.

A study by Deng *et al* found that the CC thickness increased progressively from OARSI grade 0 to grade 4 with the exception of OARSI grade 2.^38^ The authors interpreted the thinning of the CC at OARSI grade 2 as a result of early SCB plate growth evidenced by the increased irregularity of the cement line. Under normal conditions, cement line irregularity is influenced by SCB remodelling, and vascular invasion,^39^ which is increased in OA.^5 20^ In the present study, the CC was thicker in patients with OA compared with healthy subjects, and this was present also in low to moderate, that is, OARSI grade ≤3. However, the Th_CC_ declined progressively in moderate to severe OA from OARSI grade 4 to grade 6, subsequently to AC thinning and at the same stage as SCB plate thickening. Thus, our findings point towards CC thickening as a precursor for SCB plate thickening and suggest endochondral ossification and reactivation of the secondary ossification centre.^8^ This is supported by studies showing an irregularity of the cement line^8 38^ and vascularisation across the CC allowing crosstalk between the SCB plate and the AC.^36 39 40^

### Articular cartilage

As expected, the overall AC was thinner in patients with hip OA compared with healthy subjects. Reduced cartilage thickness in patients with OA was only observed in areas with OARSI grade ≥4. Our estimate of the Th_AC_ was therefore in accordance with the OARSI scoring method.^22^ Joint surface with loss of cartilage thickness (OARSI grade ≥4), however, only accounted for approximately one-fifth of the femoral head surface area in patients with OA. Radiological OA severity is determined by bone sclerosis, osteophyte formation and joint space narrowing,^31^ but as we have demonstrated, severe thickening of the SCB appears to be a marker of focal high-grade osteoarthritic lesions occurring together with complete focal loss of AC. This may partly explain the difficulties in using subchondral bone sclerosis and joint space narrowing from plain radiographs as a marker of OA disease progression.

### Strength and limitations

The present study is the first to fully apply the histopathology assessment methodology proposed by Pritzker *et al*.^22^ By the use of this technique, the entire femoral head was studied as a complete architectural object. We applied morphometric assessment of grade and stage, by combining the stereological design of systematic uniform random sampling, and vertical uniform random sections of the entire joint surface area with histological OARSI grading in the human hip OA. This method has the advantage over the OARSI score that the assessment is not biased towards the most advanced OA pathology observed.^22^ The morphometric assessment of grade and stage gives unbiased semiquantitative scores but is time consuming and requires specific expertise.

As the current study is a cross-sectional study, we were not able to directly investigate the time course of OA progression. However, if patients with OA represent subjects with a predisposition for developing OA, then regions with low-grade osteoarthritic lesions may be considered to represent early-stage OA.

In the present work, correction for tissue shrinkage during the histology processing was not performed. It has previously been demonstrated that cartilage and bone specimens from porcine hips embedded in methylmethacrylate show shrinkage of less than 2%, which can be regarded as negligible.^41^

## Conclusions

In the present cross-sectional study of human femoral heads from patients with OA and healthy subjects, we showed that the OA pathology of the CC and the SCB bone plate does not indicate progressively developing disease progression. The CC was thicker in OA, and this was present in areas without loss of AC thickness, which suggests that endochondral ossification is an early phenomenon of OA. In moderate osteoarthritic lesions, a thicker SCB plate and thinner AC further support the hypothesis that the CC may serve as a growth plate for endochondral ossification in human OA. However, significantly thickening of the SCB plate was a late-stage phenomenon of denuded bone surface only, suggesting that appositional bone growth may occur as a consequence of changed biomechanics.
