# Supplementary material for: Thickness of the bone-cartilage unit in relation to osteoarthritis severity in the human hip joint
Source: RMD Open. 2018 Sep 21;4(2):e000747. doi: 10.1136/rmdopen-2018-000747 (PMC6157564; doi:10.1136/rmdopen-2018-000747)
Supplement: Supplementary data [file rmdopen-2018-000747supp001.docx]

**Supplementary Material**

To correct the thickness measurements for oblique sectioning, we assume that the femoral head is considered as a sphere with a radius, and used the following equation:$\tilde{\mathrm{Th}_{i}}=\mathrm{Th}_{i}*sin\left( \arccos\left( \frac{\left| r- \sum_{\text{i=1}}^{\text{n}} \text{d}_{\text{i}} \right|}{r} \right) \right)$

$\tilde{Th}_{i}={Th}_{i}*sin\left( \arccos\left( \frac{\left| r- \sum_{i=1}^{n} d_{i} \right|}{r} \right) \right)$

Where *n* is the number of slices in the femoral head and *T_i_* is the measured tissue thickness, *Th_i_* is the corrected tissue thickness, and *d_i_* is the thickness of the *i*’th slice. The radius *r* of the (assumed) spherical femoral head was determined from the volume of the femoral head *V* as:

$r=\sqrt[3]{\frac{3V}{4\pi}}$


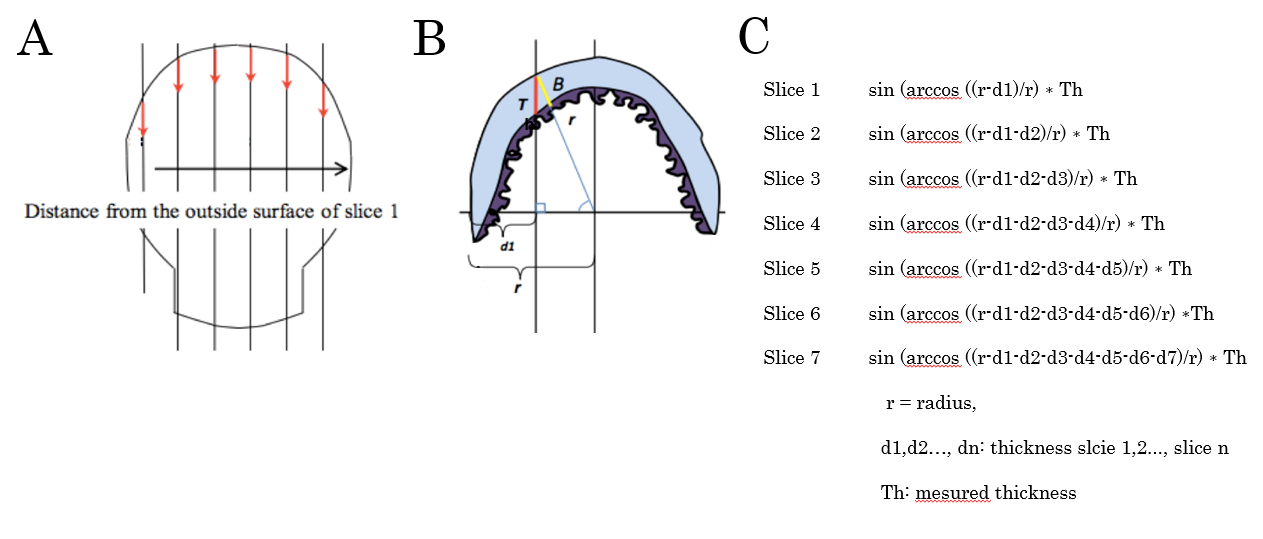


A) The femoral head was cut exhaustively using the systematic uniform random sampling and vertical uniform random sections. The black arrow indicates the direction of the measured distance from the outside of the femoral head. B) By assuming that the femoral head is spherical, a mathematical formula was made to correct for the overestimated thicknesses. C) The mathematical principles for the correction formula, where the red line ”T” is the measured thickness of AC, and the yellow line ”B” is the corrected thickness.
